# South Africans with recent pregnancy rarely know partner’s HIV serostatus: implications for serodiscordant couples interventions

**DOI:** 10.1186/1471-2458-14-843

**Published:** 2014-08-14

**Authors:** Lynn T Matthews, Lizzie Moore, Tamaryn L Crankshaw, Cecilia Milford, Fortunate N Mosery, Ross Greener, Christina Psaros, Steven A Safren, David R Bangsberg, Jennifer A Smit

**Affiliations:** Division of Infectious Disease and Center for Global Health, Massachusetts General Hospital, 100 Cambridge Street, 15th Floor, Boston, MA 02114 USA; Division of Infectious Disease, Beth Israel Deaconess Medical Center, Boston, USA; Maternal, Adolescent and Child Health (MatCH), Faculty of Health Sciences, University of the Witwatersrand, Durban, South Africa; Health Economics and HIV and AIDS Research Division, University of Kwazulu-Natal, Durban, South Africa; Department of Psychiatry, Massachusetts General Hospital, Boston, USA

**Keywords:** HIV prevention, HIV serodiscordant couples, Safer conception, HIV serostatus disclosure

## Abstract

**Background:**

Implementation of safer conception strategies requires knowledge of partner HIV-serostatus. We recruited women and men in a high HIV-prevalence setting for a study to assess periconception risk behavior among individuals reporting HIV-serodiscordant partnerships. We report screening data from that study with the objective of estimating the proportion of individuals who are aware that they are in an HIV-serodiscordant relationship at the time of conception.

**Methods:**

We screened women and men attending antenatal and antiretroviral clinics in Durban, South Africa for enrollment in a study of periconception risk behavior among individuals with serodiscordant partners. Screening questionnaires assessed for study eligibility including age 18–45 years (for women) or at least 18 years of age (for men), pregnancy in past year (women) or partner pregnancy in the past 3 years (men), HIV status of partner for recent pregnancy, participant’s HIV status, and infected partner’s HIV status having been known before the referent pregnancy.

**Results:**

Among 2620 women screened, 2344 (90%) met age and pregnancy criteria and knew who fathered the referent pregnancy. Among those women, 963 (41%) did not know the pregnancy partner’s HIV serostatus at time of screening. Only 92 (4%) reported knowing of a serodiscordant partnership prior to pregnancy. Among 1166 men screened, 225 (19%) met age and pregnancy criteria. Among those men, 71 (32%) did not know the pregnancy partner’s HIV status and only 30 (13%) reported knowing of a serodiscordant partnership prior to pregnancy.

**Conclusions:**

In an HIV-endemic setting, awareness of partner HIV serostatus is rare. Innovative strategies to increase HIV testing and disclosure are required to facilitate HIV prevention interventions for serodiscordant couples.

## Background

A large proportion of HIV-infected women and men in sub-Saharan Africa are in stable serodiscordant sexual relationships [1, 2]. As many as 30% of stable heterosexual couples in South Africa are HIV-1 serodiscordant [3, 4]. Because an estimated 50% of HIV transmission occurs between serodiscordant partners [5], these partnerships represent a priority population for prevention [6–8].

Among serodiscordant couples, antiretroviral treatment (ART) for the infected partner reduces sexual transmission by 96% [7, 9, 10] and antiretroviral pre-exposure prophylaxis (PrEP) for the uninfected partner may reduce sexual transmission by as much as 75% [11]. The World Health Organization now recommends that HIV-positive individuals with seronegative partners initiate ART to reduce sexual transmission risk [8, 12].

In South Africa, many men and women living with HIV desire children [13–15]. In the context of highly prevalent serodiscordance [3, 4], high fertility, and an absence of programs that address periconception transmission [16, 17], HIV incidence in the context of desired pregnancy is likely substantial. Antenatal clinic prevalence in KwaZulu-Natal, South Africa is estimated at 38% [18]. For serodiscordant couples who want biological children, South African guidelines recommend early ART for the infected partner in combination with other safer conception strategies, such as timed peri-ovulatory intercourse [19]. In order to implement safer conception strategies, individuals need to know their HIV status and that of their partner [20].

We recruited women and men in a high HIV-prevalence setting for a cross-sectional survey to assess periconception risk behavior among individuals reporting HIV-serodiscordant partnerships. Our study sought to enroll women with recent pregnancy and men with recent partner pregnancy. We report here our screening data with the objective of estimating the proportion of individuals who are aware that they are in a serodiscordant relationship at the time of conception.

## Methods

The study was completed in a large suburban township near Durban, South Africa. We recruited participants from antenatal care (ANC) and antiretroviral (ARV) clinics within a large hospital which serves a population of up to two million people.

All women attending ANC were systematically screened for eligibility by research assistants fluent in English and isiZulu. We sought to recruit women who were aged 18–45 and fluent in English or isiZulu; who had been pregnant in the last 12 months and knew the father’s identity; and who self-reported either a) known HIV-positive status at the time of conception with a seronegative or unknown-status pregnancy partner, or b) HIV-negative status with a seropositive pregnancy partner whose status was known before conception.

Women were encouraged to invite their male partners to participate, but due to limited recruitment, we also approached men attending the ARV clinic. Men were eligible for enrollment if they were aged 18 or older and fluent in English or isiZulu; were a partner of an enrolled female or reported partner pregnancy in the past 3 years (this longer period was used for men in order to ensure adequate recruitment); and self-reported either a) known HIV-positive status at the time of conception with a seronegative or unknown-status pregnancy partner, or b) HIV-negative status with a seropositive pregnancy partner whose status was known before conception. If men reported multiple recent pregnancy partners, we asked them to refer to the most recent pregnancy; if two or more pregnancies occurred simultaneously, we asked them to refer to the pregnancy from the most stable partnership.

Failure to meet any inclusion criterion halted the screening process. Only enrolled individuals provided identifying information. All HIV serostatus data were based on self-report by the index individual in the partnership. These screening data were collected with verbal consent; this was a screening procedure which did not collect personal identifiers.

Study data were collected and managed using REDCap electronic data capture tools hosted at Partners HealthCare [21]. All study procedures were approved by The University of the Witwatersrand Human Research Ethics Committee (Johannesburg, South Africa), the Partners (Massachusetts General Hospital) Human Research Committee (Boston, USA), Provincial and District Department of Health (eThekwini District, KwaZulu-Natal), and the local facility.

## Results

Between May 2011 and May 2012, 2620 women and 1166 men were screened for eligibility to participate in the study. Only one man was recruited via partner referral, the remainder were recruited though the ARV clinic.

**Figure 1:**
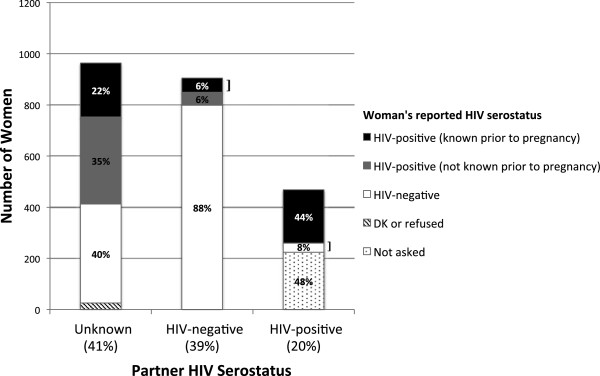
**Partner and personal HIV serostatus by self-report among 2344 women with recent pregnancy.** *Legend*: The 3 main bars show the proportion of women reporting recent pregnancy partners of unknown, HIV-negative, and HIV-positive serostatus. Within the bars, the shaded sections represent the proportion of women reporting specific HIV serostatus within those partnerships. Women in known serodiscordant relationships prior to pregnancy represent just 4% of the 2344 women screened: this group is indicated by right brackets.

Screening results for the women are shown in Figure 1. Among 2620 women, 90% (n = 2344) met age and pregnancy criteria and knew who had fathered the referent pregnancy. Among those women, 963 (41%) did not know her pregnancy partner’s current HIV serostatus, 908 (39%) reported that her partner was currently HIV-negative, and 471 (20%) reported an HIV-positive partner.

Among 963 women who did not know their partner’s status, 551 (57%) reported her own serostatus as HIV-positive but only 210 (22%) reported knowing prior to pregnancy that she was HIV-positive. 387 (40%) reported HIV-negative serostatus, and 25 (3%) did not know or refused to state her serostatus.

Among 908 women reporting an HIV-negative partner, 106 (12%) reported her own serostatus as HIV-positive but only 56 (6%) reported knowing prior to pregnancy that she was HIV-positive. 799 (88%) reported HIV-negative serostatus and 3 did not know or refused to state her serostatus.

Among 471 women reporting an HIV-positive partner, 224 (48%) reported being unaware of this prior to pregnancy and thus those women were not asked questions about their own serostatus (as in methods, failure to meet any inclusion criterion halted the screening process). Among the remainder, 208 (44% of all women reporting a currently HIV-positive partner) reported being HIV-infected and 36 (8%) reported HIV-negative serostatus.

Thus, only 92 recently pregnant women (4% of women meeting age and pregnancy criteria) reported being in a known serodiscordant partnership prior to pregnancy (depicted by right brackets in Figure 1). An additional 210 (9%) were known HIV-infected prior to pregnancy with an unknown status partner. Finally, 266 (11%) of women who met age and pregnancy criteria knew they were HIV-positive prior to pregnancy with an uninfected or unknown status partner.

**Figure 2:**
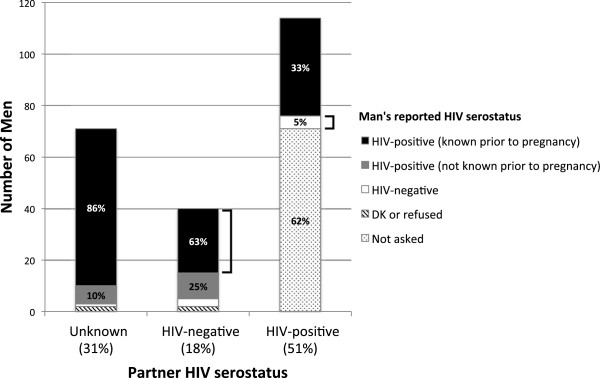
**Partner and personal HIV serostatus by self-report among 225 men with recent partner pregnancy.** *Legend*: The 3 main bars show the proportion of men reporting recent pregnancy partners of unknown, HIV-negative, and HIV-positive serostatus. Within the bars, the shaded sections represent the proportion of men reporting specific HIV serostatus within those partnerships. Men in known serodiscordant relationships prior to pregnancy represent just 13% of the 225 men screened: this group is indicated by right brackets.

Screening results for the men are shown in Figure 2. Among 1166 men screened, the majority (89%) met the age criterion, but only 225 (22% of those aged 18 and above) reported partner pregnancy in the past 3 years. We initially attempted to recruit men with partner pregnancy in the past year, but had to alter this inclusion criterion due to low recruitment (either due to low prevalence of recent partner pregnancy or low reporting thereof). Of men who met age and pregnancy criteria, 71 (32%) did not know his partner’s current HIV status, 40 (18%) reported an HIV-negative partner, and 114 (51%) reported an HIV-positive partner.

Among 71 men reporting an unknown status partner, 68 (96%) reported his own serostatus as HIV-positive and 61 (86%) reported knowing he was HIV-positive prior to pregnancy. One reported negative serostatus and 2 did not know or refused to state his HIV serostatus.

Among 40 men reporting an HIV-negative partner, 35 (88%) reported his own serostatus as HIV-positive and 25 (63%) reported knowing he was HIV-positive prior to pregnancy. Three reported HIV-negative serostatus and 2 did not know or refused to state his HIV serostatus.

Among 114 men reporting an HIV-positive partner, 71 (62%) did not know her status prior to pregnancy, thus those men were not asked questions about their own serostatus. Among the remainder, 38 (33% of all men reporting a currently HIV-positive partner) reported being HIV-infected and 5 (4%) reported HIV-negative serostatus.

Thus, a total of 30 men with recent partner pregnancy (13% of men meeting age and pregnancy criteria) reported being in a known serodiscordant partnership prior to pregnancy (depicted by right brackets in Figure 2) and an additional 61 (27%) were known HIV-infected prior to pregnancy with an unknown status partner. Finally, 86 (38%) of men who met age and pregnancy criteria, knew they were HIV-positive prior to a pregnancy with an uninfected or uknown-serostatus partner.

## Discussion

In an HIV-endemic setting, a large proportion of women (41%) and men (32%) with recent (partner) pregnancy did not know their partner’s HIV serostatus. Just 4% of 2344 women and 13% of 255 men who met age and pregnancy criteria knew that they were in a serodiscordant relationship prior to pregnancy. HIV prevalence for adults aged 15–49 years in KwaZulu-Natal is estimated at 28% [22] and the prevalence of serodiscordant couples is likely between 20 and 30% [3, 4]. Thus, our data are unlikely to reflect low prevalence of serodiscordant couples, rather they indicate the low prevalence of HIV serostatus disclosure and/or knowledge of personal HIV status. HIV prevention strategies targeting HIV-serodiscordant couples, such as early ART or PrEP, may have limited impact in South Africa without innovative solutions to increase testing and mutual disclosure between sexual partners.

Disclosure is a complex process affected by fear of stigma and discrimination [23–25], level of engagement with HIV care [26, 27], concepts of masculinity [28], communication within the relationship [29], and pregnancy itself [23, 28, 30]. Although mutual disclosure can facilitate informed decision-making and safer sex practices [26, 27], non-disclosure to sexual partners is common in South Africa [24, 26, 27, 31]. Moreover, knowledge of HIV serostatus – a first step towards serostatus disclosure – remains inadequate, especially among men [22, 32]. In our sample, 29% of HIV-positive men and 47% of HIV-positive women reporting HIV-negative partners were unaware of their own status prior to the referent pregnancy. Evidence suggests that stigma and negative attitudes towards HIV counseling and testing (HCT), as well as the physical spaces in which this is offered, continue to influence poor uptake [33–35].

To address this problem, current national [36, 37] and international [8] guidelines advocate couples-based HIV counseling and testing (CHCT). This has been associated with increased disclosure to sexual partners, enhanced adherence to interventions to reduce perinatal HIV transmission, and reductions in sexual risk behavior [38]. Yet little published data exists on CHCT in South Africa [24, 30, 39]. In a Western Cape study to promote CHCT in an ANC setting, 35% of men whose pregnant partners were given invitations for them to attend completed HCT, versus 11% of men whose partners received pregnancy education alone [40]. A behavioral intervention for pregnant women and their partners in Mpumalanga (Partnersplus) resulted in a small increase in male HIV testing and disclosure [41]. Anecdotal evidence suggests that some South African men and women use CHCT as a means of disclosure [34, 39]; however, access to and uptake of CHCT services nationally is undetermined.

Further research into CHCT is of great importance in South Africa. However, limited recruitment for couples-based interventions may forecast challenges to more widespread implementation of CHCT in South Africa: 40% of screened pregnant women could not recruit partners to the Partnersplus project [41], (D. Jones, personal communication, May 2013) and the Project Accept team screened more than three times the number of index individuals in order to interview 20 couples [42]. In our study, among 248 enrolled women, only one recruited her male partner. A ‘couple-oriented’ approach to HCT [43], which includes counseling on strategies for disclosure and to encourage partner HCT, may therefore be an important alternative in this context. This approach has demonstrated success in a recent multisite study (including one site in sub-Saharan Africa) but awaits further exploration in South Africa [43].

Although HIV prevention interventions for pregnant couples target perinatal rather than periconception transmission, their relative successes demonstrate that the desire for a healthy child can act as a powerful lever for HIV prevention. Incorporating messages on testing and mutual disclosure into PMTCT education may reduce periconception HIV transmission by moving the HIV prevention time frame ‘for a healthy baby’ up to pre-conception.

Our data suggest a high level of periconception HIV risk behavior among known HIV-positive individuals: of participants who met age and pregnancy criteria, 38% of men and 11% of women knew they were HIV-positive prior to pregnancy with an at-risk partner (pooled results for those reporting HIV-negative or unknown serostatus partners). While we do not dispute the right of these individuals to have children, many of these pregnancies were likely unintended [44–47]. There is a clear need for safer conception programs for those who choose to conceive and improved access to contraception for those who do not want to conceive [20].

These screening data represent a large sample of women and men in an HIV-endemic area with recent pregnancy. Limitations to interpretation include social desirability bias: men and women who knew their HIV status prior to pregnancy may have been reluctant to report this given counseling messages that persons living with HIV should not have sex without condoms; individuals may also have been reluctant to report partner serostatus. In addition, serodiscordance status is based on the report from just one partner. While most women were pregnant at time of screening, men were asked to reflect on a partner pregnancy in the past three years, thus recall bias may have affected responses. Additionally, since limited partner recruitment required us to enroll men from ARV clinic, male and female participants likely had very different experiences of HIV, with men more likely than women to be HIV-infected, and if so, to be engaged with treatment and support. Our screening tool stopped when any inclusion criterion was not met, thus the data are incomplete for the full sample of screened individuals.

## Conclusions

In an HIV-endemic setting, a large proportion of women (41%) and men (32%) did not know their recent pregnancy partner’s HIV serostatus. Safer conception and general HIV prevention strategies for HIV-serodiscordant couples require innovative solutions to increase testing and mutual disclosure between sexual partners. In addition, a harm-reduction approach to reducing periconception transmission should address couples as well as individuals who cannot engage their partners.
